# Evaluating Geostationary Satellite-Based Approaches for NDVI Gap Filling in Polar-Orbiting Satellite Observations

**DOI:** 10.3390/s26051731

**Published:** 2026-03-09

**Authors:** Han-Sol Ryu, Sung-Joo Yoon, Jinyeong Kim, Tae-Ho Kim

**Affiliations:** Underwater Survey Technology 21, Incheon 21999, Republic of Korea; hansol@ust21.co.kr (H.-S.R.); dryoon94@ust21.co.kr (S.-J.Y.); rlawlsdud2g@ust21.co.kr (J.K.)

**Keywords:** NDVI gap filling, geostationary satellite observations, polar-orbiting satellite, reginal vegetation monitoring, cross-orbit data fusion

## Abstract

The Normalized Difference Vegetation Index (NDVI) derived from polar-orbiting satellites is widely used for vegetation monitoring; however, its temporal continuity is often limited by cloud contamination and fixed revisit cycles. To address this limitation, this study investigates the feasibility of using geostationary satellite observations to enhance the spatial completeness of Sentinel-2 NDVI at its standard revisit intervals through cloud gap-filling applications. Geostationary Ocean Color Imager II (GOCI-II) data (250 m) was used as input, while Sentinel-2 Multispectral Instrument (MSI) NDVI (10 m) served as the reference dataset. To enable cross-sensor integration, a data-driven transformation framework was developed to convert GOCI-II NDVI into MSI-like NDVI while preserving dominant spatial variation patterns rather than pursuing strict pixel-level super-resolution. The transformed NDVI was assessed through spatial comparisons and statistical metrics, including correlation coefficient, mean absolute error, root mean square error (RMSE), normalized RMSE, and structural similarity index measure. Results show that geostationary-derived NDVI captures broad spatial organization and field-scale variability observed in MSI NDVI. Building on this cross-scale consistency, cloud gap-filling experiments demonstrate that temporally adjacent transformed NDVI scenes maintain consistent variation patterns, supporting their complementary use for compensating cloud-induced gaps. Although reduced contrast and magnitude-dependent biases remain, primarily due to the large spatial resolution difference and sub-pixel heterogeneity, an intermediate-resolution (80 m) sensitivity analysis indicates improved stability when the resolution gap is reduced. Overall, these findings highlight the practical potential of integrating geostationary and polar-orbiting observations to improve NDVI spatial continuity in cloud-prone regions.

## 1. Introduction

The Normalized Difference Vegetation Index (NDVI) is a widely used indicator in remote sensing for quantifying vegetation condition and has been extensively applied in agricultural monitoring, crop yield estimation, and vegetation change detection [1,2,3]. NDVI analysis based on optical satellite imagery is particularly effective for capturing periodic vegetation dynamics at regional scales [4], and freely available medium-resolution polar-orbiting satellites such as Sentinel-2 and Landsat are commonly utilized for this purpose. However, polar-orbiting satellite-based monitoring inherently suffers from data gaps caused by cloud contamination, as observations are acquired at fixed revisit intervals [5,6,7,8,9,10,11]. These cloud-induced missing pixels often degrade the temporal continuity of NDVI time series, making continuous monitoring of a fixed region difficult and consequently reducing the reliability of time-series-based vegetation analysis and change detection [12]. Such continuity is particularly critical for parcel-level crop condition monitoring and field-scale management and decision-support applications [13,14], where short-term variations in vegetation condition must be tracked consistently to support irrigation scheduling, fertilizer management, and crop stress assessment [15,16,17].

To address cloud-induced gaps in optical NDVI products, previous studies have proposed a range of gap-handling strategies based on different data assumptions and reconstruction mechanisms. Several studies have explored direct reconstruction through multi-sensor SAR–optical fusion, where cloud-penetrating SAR observations are integrated with optical data using machine learning or translation frameworks to recover missing vegetation information [18,19,20,21]. Other studies have employed spatiotemporal interpolation and statistical gap-filling approaches, which exploit temporal continuity and spatial correlation within optical time series to reconstruct missing pixels from neighboring observations without introducing external sensor inputs [22,23,24]. Compositing-based strategies have also been widely used to mitigate cloud contamination by selecting or aggregating cloud-free observations using quality scores or temporal compositing rules, thereby improving data availability without explicit pixel-wise reconstruction [25,26]. In addition, some studies have utilized cross-sensor complementarity and resolution differences to indirectly compensate for missing observations by integrating information from sensors with different spatial or spectral characteristics [27,28]. Although these approaches differ in their reliance on auxiliary data, temporal assumptions, and reconstruction objectives, they collectively highlight ongoing efforts to balance spatial fidelity, temporal continuity, and data availability in NDVI gap-filling applications. However, despite their usefulness in mitigating missing data, these methods remain fundamentally constrained by the revisit cycles of polar-orbiting satellites and therefore cannot fully support continuous or quasi-real-time monitoring of a fixed area. In this context, geostationary satellite observations have been suggested as a potential alternative to overcome the inherent limitations of polar-orbiting satellite-based monitoring.

Geostationary satellites acquire repeated observations of the same area within short time intervals, and because cloud positions change over time, pixels obscured by clouds at one observation time may be visible at another. In East Asia, the GEO-KOMPSAT-2B (GK-2B) satellite, launched in 2020 and carrying the Geostationary Ocean Color Imager II (GOCI-II), provides multispectral observations in the visible and near-infrared bands over regions including China, the Korean Peninsula, and Japan. GOCI-II acquires imagery at hourly intervals, enabling the capture of dense temporal observations that are difficult to obtain using polar-orbiting sensors alone [29,30]. Despite this advantage, the integration of GOCI-II with polar-orbiting satellite data for terrestrial vegetation monitoring has been limited, primarily due to its relatively coarse spatial resolution of approximately 250 m. This resolution differs substantially from that of Sentinel-2 Multispectral Instrument (MSI) (10 m) imposing significant constraints on direct comparison and gap-filling applications.

To enable geostationary observations to assist in gap filling of polar-orbiting NDVI scenes, spatial compatibility between the two datasets is essential. Because GOCI-II and MSI differ substantially in spatial resolution, resolution transformation is required to project geostationary NDVI onto the MSI grid before meaningful integration can be performed. In this sense, the transformation step functions as a technical enabler for cross-orbit data fusion aimed at restoring spatial continuity in cloud-affected NDVI observations.

Recent advances in deep learning-based spatial downscaling techniques have demonstrated strong potential for transforming coarse-resolution satellite imagery into higher-resolution products. Convolutional neural network-based models, including U-Net architectures, have shown effective performance in enhancing spatial detail while preserving large-scale patterns [31,32]. However, most existing studies focus primarily on improving the performance of downscaling algorithms themselves, with limited attention given to the practical role of geostationary satellites as reference monitoring data for continuous NDVI observation. In particular, the potential of high-resolution geostationary NDVI products as auxiliary inputs for polar-orbiting satellite NDVI gap filling has not been sufficiently explored.

To address this gap, this study evaluates the feasibility of using more frequently acquired but coarser-resolution geostationary GOCI-II NDVI observations to support spatial gap filling in higher-resolution MSI NDVI products, thereby improving the spatial completeness of vegetation monitoring in cloud-prone regions. In this framework, resolution transformation is treated as a technical prerequisite for cross-orbit integration rather than as a super-resolution objective. Given the substantial spatial scale discrepancy between the 250 m GOCI-II input and the 10 m MSI target resolution, the objective is not to reconstruct sub-pixel field-scale details, but to assess whether stable and physically consistent NDVI patterns can be generated to support more complete NDVI mapping under cloud-affected conditions. Related challenges have been addressed in previous studies through approaches aimed at improving the spatial fidelity and physical reliability of satellite imagery, including spatial resolution enhancement and reflectance correction based on physical sensor characteristics [33,34]. Other studies have focused on evaluating cross-sensor consistency and radiometric or spectral differences among satellite platforms, including spectral band adjustment, cross-calibration, and comparative analyses of vegetation indices derived from multiple sensors [35,36,37,38]. Because NDVI exhibits spatial autocorrelation and organized variability at scales larger than individual parcels, coarse-resolution observations can retain dominant vegetation structures that contribute to spatially coherent gap filling on the MSI grid.

The agricultural region of Anhui Province, China, characterized by frequent cloud cover and heterogeneous cropping patterns, was selected as the study area to evaluate the robustness of the proposed framework under realistic conditions. MSI NDVI was used as the primary data, and MSI-like NDVI generated from GOCI-II was applied to fill cloud-obscured regions. To ensure objective evaluation of gap-filling performance, quantitative validation experiments were conducted using simulated cloud masks on cloud-free MSI scenes. In addition, an intermediate-resolution experiment was designed to examine the influence of scale-gap magnitude on model stability and transformation performance. Specifically, this study first evaluates transformation performance at 10 m resolution, then examines surface-dependent stability, quantitatively validates NDVI gap filling, and finally analyzes the relationship between scale gap magnitude and transformation robustness.

## 2. Data and Study Area

In this study, GK-2B GOCI-II data and Sentinel-2A/B MSI data were used to train and test the neural network model.

GOCI-II data onboard the GK-2B satellite were used as the input data for model generation. The GK-2B satellite was launched on 18 February 2020 (UTC), and routine operations of the GOCI-II sensor began in October 2020. GOCI-II observes 12 spectral bands spanning the visible to near-infrared wavelengths from 380 to 865 nm, with an hourly temporal resolution and a spatial resolution of 250 m. In this study, Level-2 (L2) Land Surface Reflectance (SRL) data from Slot 10 of the Local Area coverage around the Korean Peninsula were used. NDVI was derived from Band 8 at 660 nm and Band 12 at 865 nm. GOCI-II L2 SRL data are provided by the National Ocean Satellite Center under the Korea Hydrographic and Oceanographic Agency and are publicly available through the official data portal, with data accessible from October 2020 to the present.

Sentinel-2 MSI data were used as the target output for model generation. Sentinel-2A was launched on 23 June 2015 (UTC), and Sentinel-2B was launched on 7 March 2017 (UTC). Data from both satellites were jointly used in this study. Each satellite has a nominal revisit cycle of 10 days, and the combined use of Sentinel-2A and Sentinel-2B enables a revisit interval of approximately 5 days. The MSI sensor consists of 13 spectral bands covering the visible to shortwave infrared wavelengths from 443 to 2190 nm, with spatial resolutions of 10, 20, and 60 m depending on the band. In this study, Level-2A (L2A) bottom-of-atmosphere (BOA) surface reflectance (SR) data from the T51SLC tile were used. NDVI was derived from Band 4 at 665 nm and Band 8 at 842 nm, both with a spatial resolution of 10 m. Sentinel-2 MSI L2A BOA SR data are publicly available through the Copernicus Data Space Ecosystem operated by the European Space Agency on behalf of the European Union’s Copernicus Programme, with data accessible from December 2018 to the present.

Table 1 summarizes the spectral bands, central wavelengths, and spatial resolutions of the satellite sensors used for NDVI computation in this study. Sentinel-2 refers to Sentinel-2A/B.

The NDVI data used in this study were calculated from the sensor-specific observations described above. NDVI was derived using a combination of a red band in the visible wavelength range around 600 nm and a near-infrared band around 800 nm. The NDVI was computed using the following equation [39]:

(1) $$NDVI=\;\frac{{SR}_{NIR}-{SR}_{RED}}{{SR}_{NIR}+{SR}_{RED}}$$

where SR denotes the surface reflectance provided by each sensor, and NIR and RED represent the near-infrared and red spectral bands, respectively. Table 2 summarizes representative NDVI values associated with major land cover categories [40], as commonly reported in previous remote sensing studies. Negative or near-zero NDVI values are typically linked to water bodies, while low positive values correspond to bare soil or sparsely vegetated surfaces. As NDVI increases, vegetation cover becomes progressively denser, with higher values indicating moderate to dense vegetation conditions. These NDVI ranges are not intended as strict classification thresholds, but rather as indicative reference values to support the interpretation of NDVI distributions in this study.

**Figure 1:**
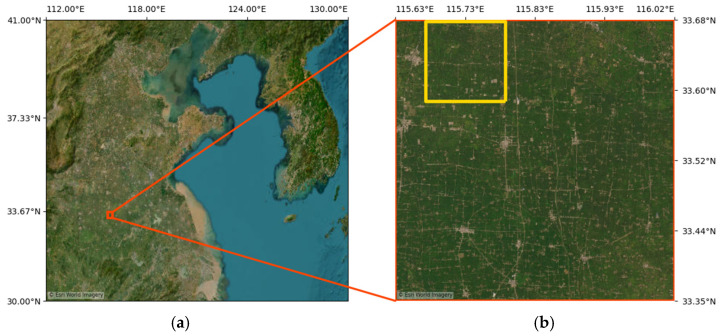
Overview of the study area: (**a**) Location of the study area within East Asia, (**b**) enlarged view showing the sub-region used for 10 m resolution analysis (yellow) and the broader area used for the intermediate-resolution experiment (orange). The background imagery is provided by Esri World Imagery.

The study area was selected over part of Anhui Province, a major agricultural region in central China characterized by extensive cropland coverage and pronounced seasonal vegetation dynamics. These characteristics provide favorable conditions for analyzing NDVI variability and for comparing geostationary satellite-derived NDVI with polar-orbiting satellite observations within a consistent observational domain. Figure 1 illustrates the geographical location of the study area. The orange boundary in Figure 1b represents the broader area used for the intermediate-resolution experiment. The yellow rectangle indicates the sub-region selected for the 10 m resolution analysis and gap-filling evaluation.

## 3. Methods

This study employed a data-driven NDVI transformation framework to convert GOCI-II NDVI into MSI-like NDVI for evaluating geostationary satellite-based approaches for NDVI gap filling. The framework was designed to address spatial inconsistencies between geostationary and polar-orbiting satellite observations while preserving physically interpretable NDVI values.

**Figure 2:**
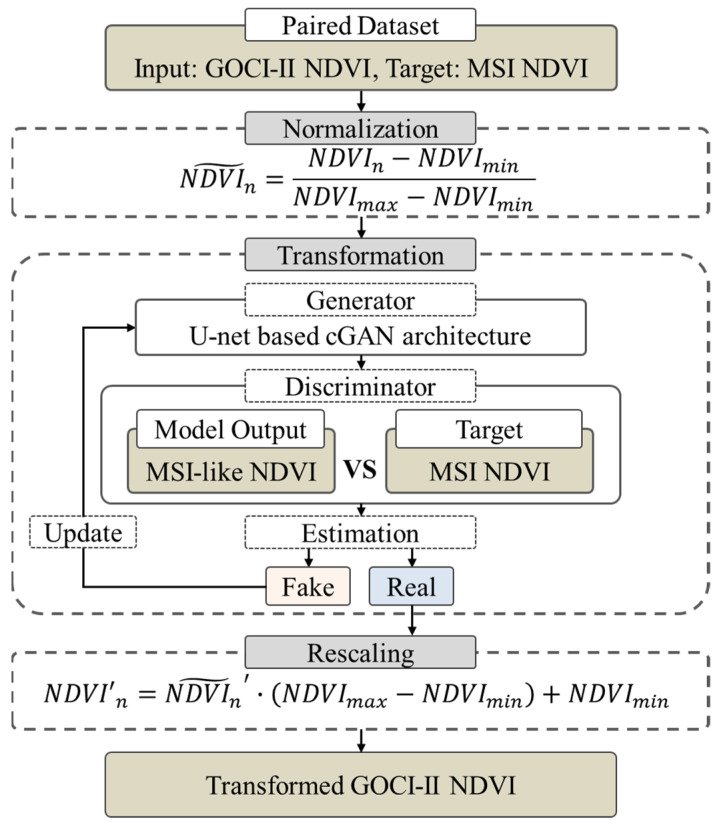
Workflow of the NDVI transformation framework. GOCI-II NDVI (input) and Sentinel-2 MSI NDVI (Target) are organized as paired datasets for model training. Through normalization, transformation, and rescaling processes, transformed NDVI is generated from GOCI-II observations.

As illustrated in Figure 2, GOCI-II NDVI (input) and MSI NDVI (Target) are first organized as paired datasets for model training. Prior to transformation, both datasets undergo normalization to ensure consistent value ranges. The normalized GOCI-II NDVI is then processed through a U-net-based conditional generative adversarial network (cGAN), consisting of a generator and a discriminator. The generator produces MSI-like NDVI (model output), which is compared with the Target MSI NDVI within the discriminator to estimate similarity and update model parameters. After transformation, the generated NDVI is rescaled back to the original NDVI range to maintain physical consistency. The final output is the transformed GOCI-II NDVI, which is subsequently used as a complementary source to improve the spatial completeness of cloud-contaminated MSI NDVI scenes. In this study, the term “transformed” is used to denote the NDVI generated through this framework under substantial spatial scale discrepancies, reflecting pattern-level consistency rather than strict pixel-wise downscaling at the MSI resolution.

The datasets used in this study cover the period from January 2023 to December 2025, encompassing the full annual cycle and associated seasonal variability in vegetation conditions. Paired NDVI cases were selected when the observation times of GOCI-II and MSI overlapped, and only scenes with less than 15% cloud coverage were retained based on the pixel-level quality flags provided in the MSI products. Pixel-level cloud masking was applied only during scene selection and not during model training. Because the MSI cloud mask is defined at 10 m resolution, directly transferring it to the 250 m GOCI-II grid would not adequately represent partial cloud contamination within coarse pixels. Such masking under substantial resolution differences could introduce inconsistencies in the paired input–target samples used for supervised learning. Following this scene-level selection approach, a total of 57 paired NDVI cases were prepared. Among these, 49 cases were used for model development, with 45 cases assigned for training and 4 cases reserved for internal validation, while the remaining 8 cases were used exclusively for independent testing. For each selected case, GOCI-II and MSI NDVI data were organized into two-dimensional arrays with a spatial size of 1020 × 1020 pixels, which were used as input–target pairs for the transformation framework.

Prior to transformation, the 250 m GOCI-II NDVI data were resampled to the 10 m MSI grid using nearest-neighbor interpolation based on geographic coordinate alignment. This procedure assigns each coarse-resolution GOCI-II pixel value to multiple 10 m MSI grid cells without introducing additional spatial detail, thereby ensuring spatial consistency across datasets while preserving the original information. NDVI values were then normalized to ensure numerical consistency and stable processing. A min–max normalization was applied based on the physical NDVI range, expressed as

(2) $$\tilde{{NDVI}_{n}}=\frac{{NDVI}_{n}-{NDVI}_{min}}{{NDVI}_{max}-{NDVI}_{min}}\;,$$

where ${NDVI}_{n}$ denotes the original NDVI value of the nth pixel for a given sensor, and ${NDVI}_{min}$ and ${NDVI}_{max}$ represent the minimum and maximum NDVI values, respectively, derived from the entire NDVI dataset of that sensor. This normalization constrained NDVI values to a range between 0 and 1 while preserving their relative distribution.

The normalized GOCI-II NDVI was transformed into MSI-like NDVI using a U-Net-based cGAN. The U-Net backbone was selected for its suitability in spatial transformation tasks and its ability to preserve multi-scale spatial patterns. Although the previous work [41,42] addressed a different application domain, both studies involve learning spatially structured mappings between heterogeneous satellite observations. In that study, a similar U-Net framework demonstrated stable performance in a hierarchical modeling setting, which informed its adoption in this feasibility study. Only the overall structural framework was conceptually retained, while model parameters and training configurations were adapted for the NDVI transformation task. The present study focuses on assessing whether the proposed transformation approach is technically feasible, using a single, consistent model architecture.

Following the transformation, the MSI-equivalent NDVI was converted back to physically interpretable NDVI values through NDVI rescaling. The rescaling process was performed using the following equation:

(3) $${NDVI^{\prime }}_{n}={\tilde{{NDVI}_{n}}}^{\prime }\cdot \left({NDVI}_{max}-{NDVI}_{min}\right)+{NDVI}_{min}\;,$$

where $NDVI_{n}^{\prime }$ represents the rescaled NDVI value on the original physical scale, and ${\tilde{{NDVI}_{n}}}^{\prime }$ denotes the normalized NDVI value transformed into MSI-equivalent NDVI.

In addition to the primary target-resolution experiment (10 m), an intermediate-resolution analysis (80 m) was conducted to examine the influence of spatial resolution discrepancy on transformation performance. The same set of paired NDVI cases described above was used to ensure consistency in temporal coverage and seasonal variability. For this analysis, the spatial domain was extended to the broader area indicated in Figure 1 (orange boundary), and both GOCI-II and MSI NDVI were resampled to an 80 m grid for transformation. This configuration maintains identical training and testing cases while isolating the effect of resolution discrepancy within a consistent experimental framework.

The rescaled NDVI from each experiment was then compared with the corresponding reference MSI NDVI to assess transformation consistency at the respective target resolutions. To quantitatively evaluate the performance of the NDVI transformation framework, pixel-level statistical validation was performed by comparing the transformed NDVI with the reference MSI NDVI. Five evaluation metrics were employed, including the Pearson correlation coefficient (R), mean absolute error (MAE), root mean square error (RMSE), normalized RMSE (nRMSE), and structural similarity index measure (SSIM). R was used to assess the linear correlation between the reference and the transformed NDVI. MAE and RMSE were used to quantify the magnitude of NDVI transformation errors, with RMSE being more sensitive to large deviations due to error squaring. nRMSE enables scale-independent comparison of relative error magnitudes. SSIM was employed to evaluate structural similarity by accounting for luminance, contrast, and spatial structure, thereby assessing the preservation of spatial patterns in NDVI fields. SSIM was computed using a sliding Gaussian window of size 11 × 11 pixels (σ = 1.5), and the final SSIM value was obtained by averaging across all valid windows. Optimal performance is characterized by R and SSIM values approaching 1, while MAE, RMSE, and nRMSE values approach 0. The equations of the four statistical metrics are provided below:

(4) $$R=\frac{\sum_{n=1}^{N}\left({NDVI}_{n}^{trans}-\bar{{NDVI}^{trans}})({NDVI}_{n}^{ref}-\bar{{NDVI}^{ref}}\right)}{\sqrt{\sum_{n=1}^{N}{\left({NDVI}_{n}^{trans}-\bar{{NDVI}^{trans}}\right)}^{2}}\sqrt{\sum_{n=1}^{N}{\left({NDVI}_{n}^{ref}-\bar{{NDVI}^{ref}}\right)}^{2}}}\;,$$

(5) $$MAE=\frac{1}{N}\sum_{n=1}^{N}\left|{NDVI}_{n}^{trans}-{NDVI}_{n}^{ref}\right|\;,$$

(6) $$RMSE=\sqrt{\frac{1}{N}\sum_{n=1}^{N}{\left({NDVI}_{n}^{trans}\right.-\left.{NDVI}_{n}^{ref}\right)}^{2}}\;,$$

(7) $$nRMSE=\frac{RMSE}{{NDVI}_{max}-{NDVI}_{min}}\;\;,$$

(8) $$SSIM=\frac{\left(2\mu_{trans}\mu_{ref}+C_{1})(2\sigma_{trans,ref}+C_{2}\right)}{\left(\mu_{trans}^{2}+\mu_{ref}^{2}+C_{1})(\sigma_{trans}^{2}+\sigma_{ref}^{2}+C_{2}\right)}$$

where $NDVI_{n}^{trans}$ denotes the transformed NDVI value of the nth pixel, $NDVI_{n}^{ref}$ denotes the corresponding reference MSI NDVI, and N is the total number of pixels used for evaluation. ${NDVI}_{max}$ and ${NDVI}_{min}$ represent the theoretical maximum and minimum values of NDVI, respectively (−1 and 1). μ and σ represent the mean and variance of each NDVI dataset, respectively, $\sigma_{trans,ref}$ denotes the covariance between transformed and reference NDVI $C_{1}$ and $C_{2}$ are small constants introduced to stabilize the SSIM computation, following the standard SSIM formulation.

## 4. Results

### 4.1. Comparison Between Transformed GOCI-II NDVI and Reference MSI NDVI

This section presents a comparative analysis between the transformed NDVI derived from GOCI-II and the reference MSI NDVI at 10 m spatial resolution. The analysis examines the extent to which geostationary-derived NDVI maintains spatial structure and vegetation variability when represented on the MSI grid. Spatial correspondence and quantitative agreement are evaluated to assess cross-scale consistency in preparation for subsequent MSI NDVI gap-filling applications.

**Figure 3:**
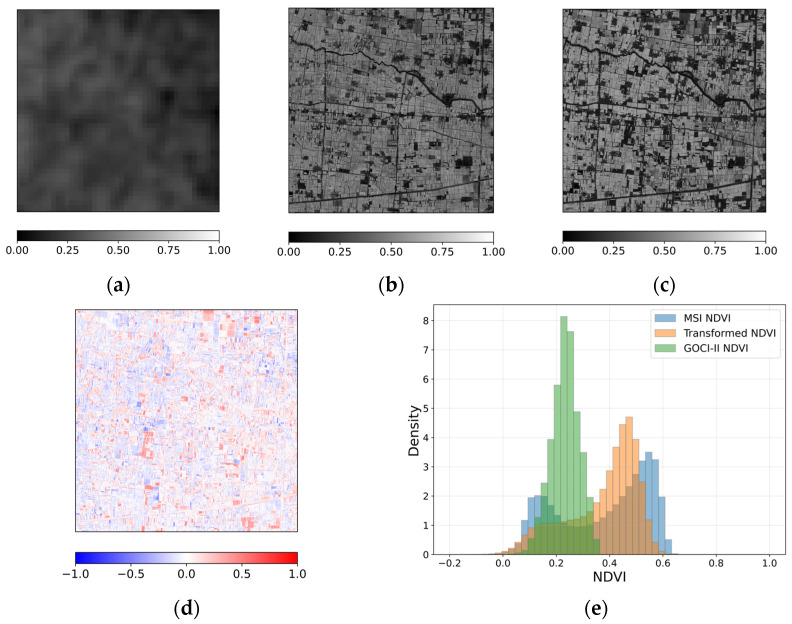
Spatial comparison of NDVI for the case acquired on 13 January 2025: (**a**) GOCI-II NDVI, (**b**) transformed NDVI, (**c**) reference MSI NDVI, (**d**) difference between transformed NDVI and reference MSI NDVI, and (**e**) histogram illustrating the NDVI value distributions of GOCI-II, transformed NDVI and reference MSI NDVI.

Figure 3 presents a spatial comparison of NDVI derived from different sensors and the transformed result for a representative case acquired on 13 January 2025 over the study area. Figure 3a shows the original GOCI-II NDVI, which exhibits relatively smooth spatial patterns due to its coarse spatial resolution. Fine-scale land parcel boundaries and linear features are not clearly discernible, reflecting the inherent spatial limitation of GOCI-II NDVI. When represented on the MSI grid, the transformed NDVI (Figure 3b) shows spatial patterns that are more consistent with those observed in reference MSI NDVI (Figure 3c). Field-scale variations and agricultural structures become distinguishable compared to the original GOCI-II NDVI. Rather than reproducing fine-scale detail, the transformation enables alignment of dominant vegetation patterns with the spatial framework of the MSI observation. The difference map between the transformed NDVI and MSI NDVI (Figure 3d) reveals both positive and negative deviations distributed across the scene. Differences occur at parcel boundaries as well as within agricultural fields, reflecting the influence of spatial resolution discrepancy and sub-pixel heterogeneity. The deviations appear spatially organized rather than randomly scattered, indicating that the transformation maintains overall NDVI pattern continuity while introducing localized differences.

The histogram comparison (Figure 3e) further illustrates the NDVI value distributions of the three NDVI datasets. Overall, the transformed NDVI follows the distribution of MSI NDVI more closely than the original GOCI-II NDVI, particularly within the dominant mid-range vegetation values. However, in the lower NDVI range, MSI exhibits a greater proportion of pixels and a peak located at slightly lower values compared to the transformed NDVI, indicating that low NDVI conditions are not fully represented in the transformed distribution. Although slight differences in magnitude are observed, the overall scene-level distribution characteristics remain broadly comparable. The scene-level mean NDVI values are 0.233 for GOCI-II, 0.374 for the transformed NDVI, and 0.375 for MSI NDVI. The corresponding standard deviations are 0.052, 0.133, and 0.171, respectively. The transformed NDVI therefore closely matches the MSI mean while exhibiting intermediate variability between the coarse-resolution GOCI-II and the reference MSI NDVI.

For the case acquired on 13 January 2025, the statistical comparison between the transformed NDVI and the reference MSI NDVI yielded an R value of 0.589. The MAE and RMSE values were 0.108 and 0.142, respectively, and the nRMSE was 0.071. The SSIM value was 0.494. These values indicate moderate pixel-level agreement between the transformed and reference NDVI, with discrepancies remaining at finer spatial scales.

**Figure 4:**
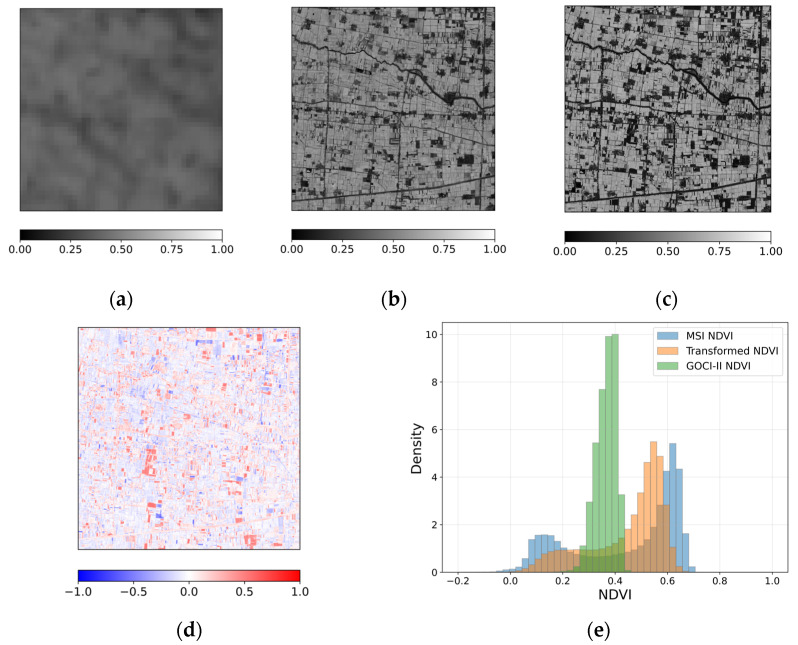
Spatial comparison of NDVI for the case acquired on 19 March 2025: (**a**) GOCI-II NDVI, (**b**) transformed NDVI, (**c**) reference MSI NDVI, and (**d**) difference between transformed NDVI and reference MSI NDVI, and (**e**) histogram illustrating the NDVI value distributions of GOCI-II, transformed NDVI and reference MSI NDVI.

Figure 4 presents the spatial comparison of NDVI for the case acquired on 19 March 2025. The transformed NDVI (Figure 4b) shows increased spatial differentiation relative to the original GOCI-II NDVI (Figure 4a) and exhibits general correspondence with the reference MSI NDVI shown in Figure 4c. Field-scale variability and major linear landscape features are discernible, indicating that the transformation aligns dominant NDVI patterns with those observed in the MSI. The difference map between the transformed NDVI and the reference MSI NDVI (Figure 4d) reveals spatially structured deviations distributed across the scene. Positive and negative differences occur in clustered patches at the field scale, indicating that discrepancies follow organized spatial patterns rather than random pixel-level noise. While localized discrepancies remain evident, large-scale systematic bias is not apparent. Overall, the transformed NDVI maintains broad spatial organization observed in MSI NDVI, with difference primarily occurring finer spatial scales.

The histogram comparison in Figure 4e shows a distribution pattern similar to that observed in Figure 3e. The transformed NDVI shifts toward the MSI NDVI distribution relative to the original GOCI-II NDVI, while differences persist in the lower NDVI range where MSI includes a greater proportion of low values. The scene-level mean NDVI values are 0.365 for GOCI-II, 0.446 for the transformed NDVI, and 0.441 for MSI NDVI. The corresponding standard deviations are 0.038, 0.145, and 0.205, respectively. The transformed NDVI closely matches the MSI mean and exhibits substantially increased variability relative to GOCI-II, while remaining lower than the MSI dispersion.

For the case acquired on 19 March 2025, the comparison between the transformed NDVI and the reference MSI NDVI yielded R = 0.647, MAE = 0.116, RMSE = 0.157, nRMSE = 0.079, and SSIM = 0.515. These values indicate moderate pixel-level agreement, with differences remaining at finer spatial scales.

Table 3 summarizes the statistical evaluation results for the eight independent test cases. Across all cases, the average R was 0.511, while the MAE and RMSE were 0.108 and 0.139, respectively. The average nRMSE was 0.070, and the mean SSIM was 0.514. These results indicate consistent pixel-level correspondence across multiple seasonal conditions, with variability observed among individual dates. In most cases, R values range between approximately 0.47 and 0.65, while nRMSE remains below 0.08, indicating stable relative error magnitude across the test set. The case acquired on 21 November 2025 shows comparatively lower correlation (R = 0.216) while maintaining moderate structural similarity (SSIM = 0.520). On this date, both GOCI-II and MSI observations exhibited uniformly low NDVI values across the scene. This case is therefore considered a low-contrast condition in the input observations, representing a data-specific scenario rather than a structural limitation of the transformation framework. The moderate agreement observed across the test cases reflects the substantial native resolution difference and the single-stage transformation framework.

**Figure 5:**
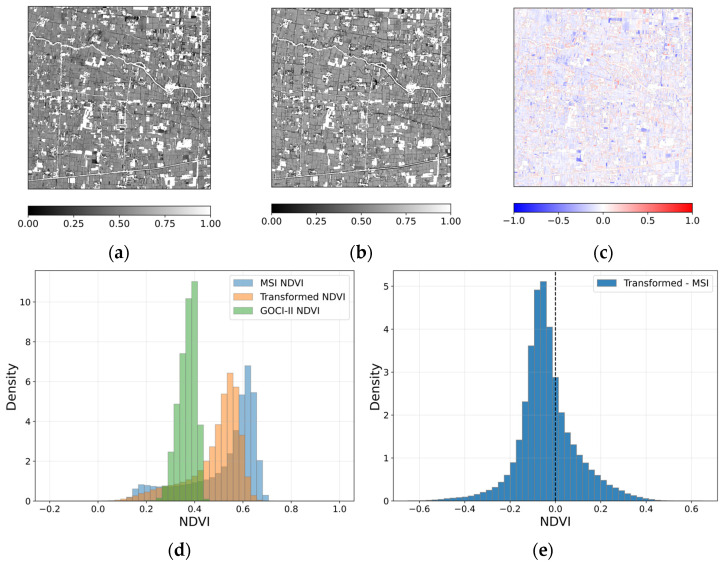
NDVI comparison over vegetated areas (non-vegetated regions excluded) for the case acquired on 19 March 2025: (**a**) transformed NDVI, (**b**) reference MSI NDVI, (**c**) difference between transformed NDVI and MSI NDVI, (**d**) histogram comparing distributions of GOCI-II NDVI, transformed NDVI, and MSI NDVI, and (**e**) histogram of the NDVI differences shown in (**c**).

To further examine the influence of land-cover characteristics on transformation behavior, an additional analysis focusing exclusively on vegetated areas was conducted for the 19 March case. Vegetated regions were defined using temporally aggregated MSI NDVI data from 2025, with pixels having mean NDVI values ≤ 0.25 classified as non-vegetated based on the indicative threshold provided in Table 2. The comparison results are presented in Figure 5. Figure 5a,b show the transformed NDVI and reference MSI NDVI over vegetated areas only, while Figure 5c presents their difference after excluding non-vegetated regions. Compared with the unmasked results (Figure 4), the spatial deviations appear more spatially balanced when non-vegetated areas are removed. Differences remain distributed at the field scale, but large coherent overestimation patterns are less evident. The histogram comparison in Figure 5d further illustrates the NDVI distributions over vegetated areas. The transformed NDVI exhibits a distribution that is closer to MSI NDVI than to the original GOCI-II NDVI, particularly within the dominant vegetation range. The lower-NDVI portion of the distribution is substantially reduced after masking, resulting in improved alignment between the transformed and reference NDVI.

Figure 5e shows the histogram of the NDVI differences (transformed minus MSI) over vegetated areas. The distribution is slightly shifted toward negative values, indicating a tendency for underestimation in vegetated regions. Most differences are concentrated within a moderate range, and extreme deviations are limited. This negative shift is also reflected in the NDVI distribution comparison shown in Figure 5d, where the transformed NDVI exhibits slightly lower values than MSI within the dominant vegetation range. The histogram of the NDVI difference shows a mean of −0.036 and a standard deviation of 0.126. The negative mean indicates a slight overall underestimation in the transformed NDVI, while the dispersion reflects moderate variability of differences around zero.

**Figure 6:**
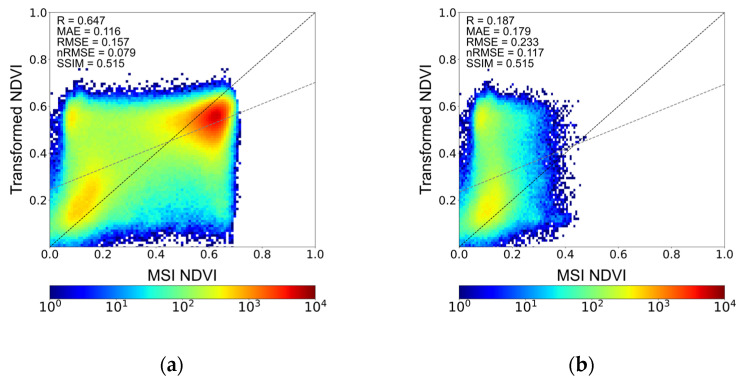
Density scatter plots comparing transformed NDVI and MSI NDVI for (**a**) the full study area and (**b**) non-vegetated regions on 19 March 2025.

Figure 6 illustrates the distributional relationship between the transformed NDVI and the reference MSI NDVI using density scatter plots. Figure 6a shows the results over the entire study area, while Figure 6b presents the distribution restricted to non-vegetated regions. In Figure 6a, the transformed NDVI exhibits magnitude-dependent deviations relative to MSI NDVI. Lower NDVI values tend to be slightly overestimated, whereas higher NDVI values show a tendency toward underestimation. When the analysis is restricted to non-vegetated regions (Figure 6b), the overestimation at low NDVI values becomes more pronounced, indicating that this behavior is primarily associated with low-NDVI surfaces. In contrast, the underestimation observed at higher NDVI values remains evident in the full-area comparison and is not explained solely by non-vegetated regions. This pattern reflects the challenges associated with directly transforming NDVI across a substantial spatial resolution discrepancy using a single-stage framework, where fine-scale high-NDVI variability captured by MSI may not be fully represented in the transformed results.

### 4.2. Application of Transformed GOCI-II NDVI for Cloud Gap Filling in MSI NDVI

**Figure 7:**
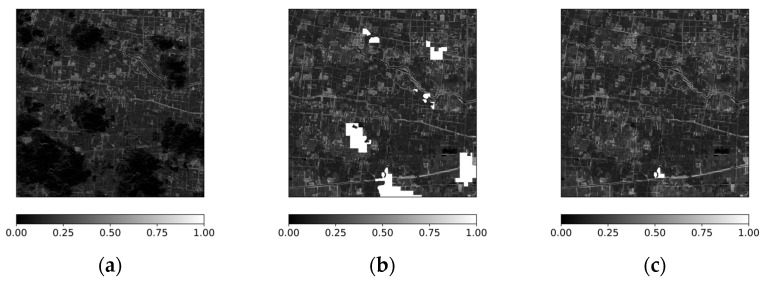

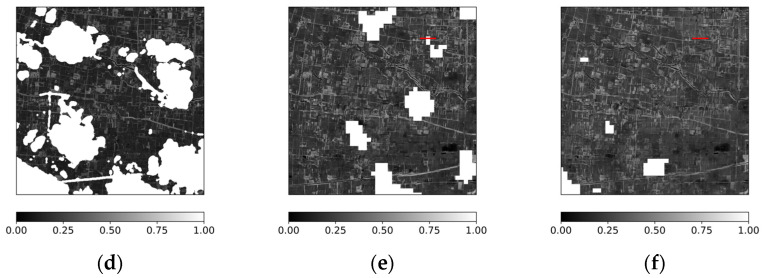
Example of NDVI gap filling on 28 May 2025. (**a**) Cloud-obscured MSI NDVI at 02:55 UTC; (**b**) gap-filled MSI NDVI using transformed NDVI at 03:15 UTC; (**c**) gap-filled MSI NDVI using transformed NDVI at 03:15 and 02:15 UTC; (**d**) cloud-masked MSI NDVI; (**e**) cloud-masked transformed NDVI at 03:15 UTC; (**f**) cloud-masked transformed NDVI at 02:15 UTC. The red lines indicate transects within the cloud-induced gap used for NDVI comparison.

To examine the applicability of the transformed geostationary NDVI for supporting cloud gap filling in polar-orbiting satellite observations, the transformed NDVI was applied to MSI NDVI scenes affected by cloud contamination, as identified using the cloud quality information provided in the MSI products [43]. Figure 7 presents an example acquired on 28 May 2025 at 02:55 UTC, in which substantial cloud-induced data gaps are present in the MSI NDVI (Figure 7a).

As a first step, the missing regions were filled using the temporally closest transformed NDVI acquired at 03:15 UTC, after excluding cloud-contaminated pixels based on the GOCI-II cloud flag information [44]. The resulting gap-filled MSI NDVI is shown in Figure 7b. A substantial portion of the cloud-affected areas were filled; however, some gaps remained due to incomplete spatial overlap between the MSI and transformed NDVI scenes. To further reduce the remaining gaps, an additional gap-filling step was performed using the transformed NDVI acquired at 02:15 UTC, producing the composite result shown in Figure 7c. Compared with the original cloud-obscured MSI NDVI (Figure 7a), spatial continuity is improved and discontinuities along the cloud boundaries are reduced. Figure 7d and Figure 7f present the cloud-masked MSI NDVI at 02:55 UTC and the cloud-masked transformed NDVI at 03:15 and 02:15 UTC, respectively. Overall, this example demonstrates that sequential use of temporally adjacent transformed NDVI scenes can progressively reduce cloud-induced gaps and enhance the spatial completeness of MSI NDVI.

**Figure 8:**
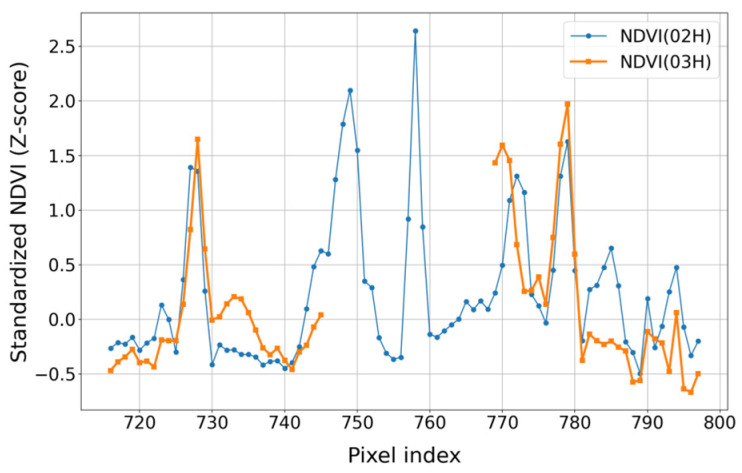
Standardized NDVI profile comparison along the red transects shown in Figure 7e,f for 02:15 and 03:15 UTC on 28 May 2025.

To further examine whether the filled regions maintain consistent spatial variation patterns across adjacent acquisition times, a profile-based comparison was conducted. Because the focus of this analysis is on variation structure rather than exact pixel-level agreement, the transformed NDVI profiles were compared after applying z-score normalization. The red lines in Figure 7e,f indicate a transect located within a cloud-induced gap in the MSI scene at 02:55 UTC, where the 03:15 observation is affected by cloud contamination while the 02:15 observation provides valid coverage. Figure 8 presents the standardized NDVI profiles along this transect for the transformed NDVI at 02:15 and 03:15 UTC. The blue and orange curves correspond to the two acquisition times, respectively. Although differences in magnitude are observed, the overall variation patterns along the pixel sequence remain broadly consistent between the two times. Discontinuities appear in the 03:15 profile where cloud effects are present, whereas the 02:15 profile maintains a continuous trend across the same region. This comparison indicates that temporally adjacent transformed NDVI scenes exhibit comparable spatial variation structures and can be used complementarily to compensate for cloud-induced gaps in MSI NDVI. Such applicability is particularly relevant in contexts where field-scale NDVI patterns and spatial completeness are prioritized over strict pixel-wise agreement, including parcel-level crop condition assessment and management-oriented agricultural monitoring.

**Figure 9:**
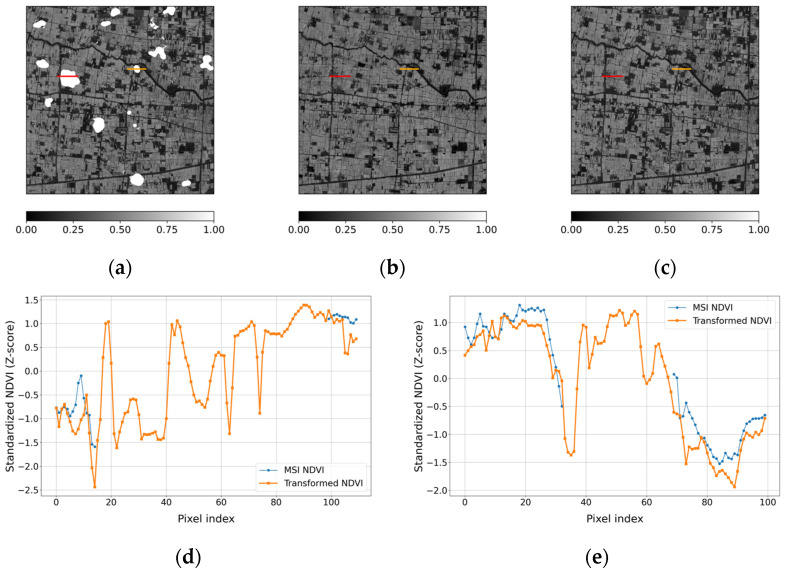
Simulated cloud-mask gap-filling experiment for the case acquired on 27 February 2025. (**a**) MSI NDVI with artificially masked cloud regions; (**b**) transformed NDVI; (**c**) gap-filled MSI NDVI using the transformed NDVI; The red and orange lines indicate transects used for profile-based analysis. (**d**) Standardized NDVI profiles along the red transect, and (**e**) corresponding profiles along the orange transect, comparing MSI NDVI and transformed NDVI.

To further evaluate the applicability of the transformed NDVI for gap filling, a simulated cloud-mask experiment was conducted for the case acquired on 27 February 2025 (Figure 9). Artificial cloud regions were generated in the MSI NDVI (Figure 9a), and the masked areas were subsequently filled using the transformed NDVI (Figure 9c). Spatially, the gap-filled result preserves the dominant NDVI patterns observed in the original MSI scene, without introducing abrupt discontinuities along the masked boundaries. The transect-based comparison (Figure 9d,e) further indicates that the transformed NDVI follows the general variation trends of MSI NDVI along both profiles. Although local discrepancies are present, the overall pattern alignment remains consistent across the transects. A quantitative comparison over the artificially masked regions yields R = 0.656, MAE = 0.088, RMSE = 0.121, and nRMSE = 0.061, indicating moderate agreement in magnitude while maintaining consistent variation structure. These results suggest that the transformed NDVI captures the primary spatial variation structure of MSI NDVI and can serve as auxiliary information for compensating cloud-induced gaps. The remaining discrepancies in magnitude reflect the structural limitation associated with applying a single-stage transformation across a substantial spatial resolution difference. This observation motivates further examination of scale-gap effects, which is addressed in the following section through an intermediate-resolution sensitivity analysis.

### 4.3. Scale-Gap Sensitivity Analysis Using Intermediate Resolution

An additional transformation experiment was conducted at an intermediate spatial resolution of 80 m to examine the influence of spatial scale on transformation behavior. In this setup, MSI NDVI was aggregated to 80 m, and GOCI-II NDVI was resampled to the same 80 m grid prior to model construction. To secure sufficient training samples at this resolution, the analysis domain was expanded to the broader region shown in Figure 1b, and image patches of 300 × 300 pixels were generated using a sliding-window approach with one-third overlap.

**Figure 10:**
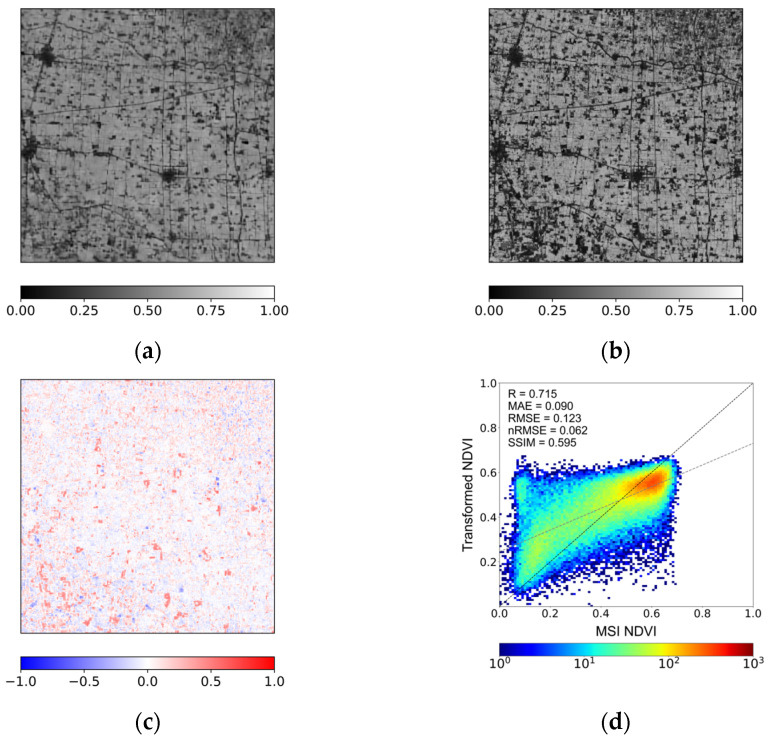
Comparison of NDVI transformation at an intermediate spatial resolution (80 m) for the 19 March 2025 case, showing (**a**) transformed NDVI, (**b**) reference MSI NDVI, (**c**) difference between transformed NDVI and MSI NDVI, and (**d**) the corresponding density scatter plot.

Figure 10 presents the intermediate-resolution (80 m) transformation results for the case acquired on 19 March 2025, corresponding to the same date analyzed in Figure 4 and Figure 5. The transformed NDVI (Figure 10a) and the MSI NDVI resampled to 80 m (Figure 10b) exhibit comparable large-scale spatial distributions. The difference map (Figure 10c) shows that spatially organized deviations remain; however, the magnitude of these deviations appears reduced relative to the direct 10 m transformation. The density scatter plot in Figure 10d provides a clearer indication of this behavior. Compared with the 10 m case (Figure 4), the samples at 80 m are more tightly concentrated along the 1:1 line. The spread of points is reduced, particularly in the higher NDVI range, where underestimation is less pronounced than in the direct 10 m transformation. Although slight overestimation at low NDVI values persists, the overall dispersion is more compact, indicating improved consistency between the transformed and reference NDVI at the intermediate resolution.

These results show that when the spatial resolution gap between input and target grids is reduced, the transformation behavior becomes more stable in terms of value distribution and sample dispersion. The intermediate-resolution experiment therefore suggests that constraining the transformation to a more moderate scale may alleviate part of the mismatch observed under the larger resolution discrepancy. In this context, a multi-stage transformation strategy, which progressively bridges the resolution gap, may provide a more stable pathway for utilizing geostationary NDVI as auxiliary information for polar-orbiting satellite gap filling.

## 5. Discussion

This study examined the feasibility of transforming geostationary GOCI-II NDVI to support gap filling in polar-orbiting MSI NDVI. The results demonstrate that, despite the substantial spatial resolution discrepancy between the two sensors, the transformed NDVI consistently preserves dominant spatial organization and relative vegetation variability at the field scale. Across multiple independent test cases, moderate but stable statistical agreement was observed, indicating reproducible cross-scale correspondence under varying seasonal and surface conditions.

It is important to emphasize that the objective of this study was not to achieve pixel-level super-resolution reconstruction of MSI NDVI. Instead, the transformation serves as a technical mechanism to establish spatial compatibility between heterogeneous satellite observations. In this context, the moderate statistical performance should be interpreted in light of the inherent structural constraint imposed by the 250 m to 10 m resolution gap. Fine-scale variability captured at 10 m cannot be fully recovered when it is not explicitly represented in the coarse-resolution geostationary observations. Therefore, the observed magnitude-dependent deviations, such as slight overestimation at low NDVI and underestimation at higher NDVI levels, reflect intrinsic cross-scale limitations rather than purely model-related deficiencies.

This structural constraint is further influenced by the characteristics of the study region. The agricultural landscape is composed of relatively small and heterogeneous fields, often involving mixed cropping practices. At 250 m resolution, individual GOCI-II pixels inevitably aggregate multiple crop types and phenological stages, amplifying mixed-pixel effects. Under such conditions, perfect pixel-level correspondence with 10 m MSI observations is not physically attainable. The transformation results therefore represent the upper bound of cross-scale representation achievable under a single-stage approach across a large resolution gap.

The intermediate-resolution experiment provides additional insight into the role of spatial resolution disparity in transformation stability. When both datasets were harmonized at 80 m resolution, the density scatter plot exhibited improved concentration along the 1:1 line and reduced magnitude bias compared with the direct 10 m transformation. Although structured deviations remained, their amplitude decreased. This behavior indicates that the magnitude of the scale gap plays a critical role in transformation stability. As the resolution discrepancy narrows, the model is required to infer fewer sub-grid spatial variations, leading to improved consistency in NDVI magnitude representation.

Importantly, the improved behavior at 80 m does not contradict the 10 m objective of this study. Rather, it suggests that progressively bridging the resolution gap may provide a more stable pathway for cross-scale NDVI transformation. In this context, a multi-stage transformation strategy, where NDVI is sequentially refined through intermediate spatial resolutions, may reduce resolution-induced biases while maintaining physical consistency with the information content of geostationary observations. This possibility warrants further investigation in future work.

From an application perspective, the relevance of this transformation should be interpreted within the broader objective of NDVI gap filling. The gap-filling experiments demonstrate that temporally adjacent transformed NDVI scenes can progressively reduce cloud-induced discontinuities in MSI observations. Profile-based analyses further confirm that spatial variation patterns remain broadly consistent across temporally adjacent geostationary observations, even when absolute magnitude differences persist. In cloud-affected scenes, the preservation of coherent field-scale patterns and spatial continuity is often more critical than strict pixel-wise agreement. Within this operational context, the transformed NDVI provides meaningful auxiliary information for enhancing the spatial completeness of MSI NDVI under cloudy conditions.

Compared with conventional NDVI gap-filling approaches relying solely on temporal interpolation of polar-orbiting satellite observations [45,46], the present framework incorporates complementary frequent information from geostationary sensors. By enabling spatial alignment through cross-scale transformation, the approach extends the methodological scope of NDVI gap filling beyond revisit-cycle-constrained interpolation. Although this study focuses on GOCI-II observations over East Asia, the framework is conceptually transferable to other geostationary platforms, subject to spectral compatibility and regional coverage.

Overall, the findings confirm both the potential and structural limitations of reconstructing polar-orbiting NDVI using geostationary observations. While fine-scale pixel-level information cannot be fully recovered under a large single-stage resolution gap, meaningful spatial organization and temporal complementarity can be achieved. The intermediate-resolution analysis further indicates that transformation robustness improves as the scale gap decreases, suggesting that progressive cross-scale refinement may enhance stability. These results establish a technical foundation for geostationary-assisted NDVI gap filling and highlight cross-orbit data fusion as a promising direction for improving the spatial completeness and operational usability of high-resolution NDVI products in agricultural monitoring applications.

## 6. Conclusions

This study explored the feasibility of using geostationary satellite observations to support NDVI transformation for polar-orbiting satellite data, with the primary objective of enhancing the spatial completeness of MSI NDVI observations at their standard revisit intervals by utilizing geostationary satellite data as a complementary source. By integrating GK-2B GOCI-II observations (250 m) with Sentinel-2 MSI NDVI (10 m) as the primary reference, the results demonstrate that geostationary data can be effectively utilized to reproduce broad spatial NDVI patterns at MSI-like spatial resolution (10 m) over fixed regions, despite inherent spatial resolution differences. Rather than focusing on precise pixel-level transformation, this work emphasizes a monitoring framework in which high-resolution polar-orbiting satellite data provide spatial detail, while geostationary satellite observations serve as a complementary source for reducing cloud-induced gaps and improving the spatial completeness of high-resolution NDVI scenes. The high temporal sampling capability of geostationary satellites offers additional observation opportunities that may assist NDVI transformation under cloud-contaminated conditions, thereby extending the practical usability of MSI NDVI products.

Although the transformation results reveal structural limitations associated with the large spatial resolution gap, the analysis also confirms that dominant NDVI spatial organization and field-scale variability are preserved to a meaningful extent. Furthermore, the intermediate-resolution sensitivity experiment indicates that transformation stability improves as the resolution gap decreases, suggesting that progressive or multi-stage cross-scale refinement may provide a more robust pathway for future enhancement. Overall, this study suggests that integrating polar-orbiting and geostationary satellite observations provides a practical and conceptually consistent strategy for improving the spatial completeness and operational usability of high-resolution NDVI products in cloud-prone regions. The proposed framework establishes a foundation for future refinement of geostationary-polar orbit integrated NDVI transformation and its application to continuous agricultural and regional vegetation monitoring.

## Figures and Tables

**Table 1:** Spectral band characteristics of the GK-2B GOCI-II and Sentinel-2 MSI sensors used in this study.

Sensor	Band	Central Wavelength (nm)	Resolution (m)
GK-2B GOCI-II	Band 8	660	250
	Band 12	865	
Sentinel-2 MSI	Band 4	665	10
	Band 8	842	

**Table 2:** Representative NDVI values used to characterize different land cover types.

Land Cover Types	NDVI Threshold
Water	−0.046
Bare soil	0.25
Sparse vegetation	0.35
Moderate vegetation	0.5
Dense vegetation	1.0

**Table 3:** Statistical metrics for quantitative comparison between transformed NDVI and MSI NDVI.

Date (2025)	R	MAE	RMSE	nRMSE	SSIM
13 January	0.589	0.108	0.142	0.071	0.494
18 January	0.528	0.121	0.155	0.078	0.467
28 January	0.575	0.111	0.143	0.072	0.507
27 February	0.640	0.086	0.119	0.060	0.571
19 March	0.647	0.116	0.157	0.079	0.515
28 April	0.423	0.139	0.161	0.081	0.487
24 July	0.472	0.086	0.113	0.057	0.547
21 November	0.216	0.099	0.126	0.063	0.520
Average	0.511	0.108	0.139	0.070	0.514
Standard Deviation	0.142	0.018	0.018	0.009	0.033

## Data Availability

The original contributions presented in this study are included in the article. Further inquiries can be directed to the corresponding author.

## Abbreviations

The following abbreviations are used in this manuscript:

BOA	Bottom-Of-Atmosphere
cGAN	Conditional Generative Adversarial Network
GK-2B	GEO-KOMPSAT-2B
GOCI-II	Geostationary Ocean Color Imager-II
L2	Level-2
L2A	Level-2A
MAE	Mean Absolute Error
MSI	Multispectral Instrument
NDVI	Normalized Difference Vegetation Index
nRMSE	Normalized Root Mean Square Error
R	Pearson Correlation Coefficient
RMSE	Root Mean Square Error
SR	Surface Reflectance
SRL	Land Surface Reflectance
SSIM	Structural Similarity Index Measure
USGS	United States Geological Survey
